# Ampullary Adenocarcinoma Causing Small Intestinal Obstruction

**DOI:** 10.7759/cureus.11575

**Published:** 2020-11-19

**Authors:** Mohammad Abudalou, Michael Malkowski, Alex Huh, David Ricklan, Christopher Stallwood

**Affiliations:** 1 Medicine, St. Elizabeth's Medical Center, Brighton, USA; 2 Gastroenterology, Tufts Medical Center, Boston, USA; 3 Pathology, St. Elizabeth's Medical Center, Brighton, USA; 4 Gastroenterology, St. Elizabeth's Medical Center, Brighton, USA

**Keywords:** gastrointestinal obstruction, ampullary adenocarcinoma, whipple procedure, ct (computed tomography) imaging

## Abstract

Ampullary adenocarcinoma is a malignant tumor that arises from the ampullary complex, distal to the confluence of common bile duct and pancreatic duct. It is a rare tumor and pathologically differentiated into intestinal or pancreaticobiliary in origin. Management is surgical resection. We report a case of a 67-year-old male who presented with abdominal pain, vomiting, and constipation. Computed tomography scan showed a cystic mass compressing the duodenum and causing small intestinal obstruction. Pathologic evaluation was consistent with ampullary adenocarcinoma.

## Introduction

Ampullary adenocarcinoma is a rare malignant tumor that constituting only 0.2% of all gastrointestinal tumors [1]. These tumors usually arise from preexisting adenomas or flat preneoplastic lesions. More than 95% of benign ampullary neoplasms are adenomas of intestinal type and have a tubular, villous or mixed tubulovillous pattern resembling adenomas of the intestine [2]. Jaundice is the most common presenting symptom exhibited in 82% of patients while dyspepsia, malaise, and anorexia occur less frequently, in 15%-30% of cases [3]. These tumors are detected on radiologic imaging and in 62% of cases they appear as a nodular solid mass [4]. Definitive diagnosis is obtained from pathologic evaluation after surgical resection which is the mainstay of treatment. The prognosis after surgical resection is favorable and the overall one, two, and five-year survival rate is 99%, 96%, and 86% respectively [5].

## Case presentation

A 67-year-old male patient with a history of tobacco use and alcohol dependence presented with two weeks of abdominal pain, distention, vomiting, and a 30-pound weight loss over the course of three months. On presentation, he was afebrile with normal blood pressure and heart rate. On exam, he appeared cachectic, with left upper quadrant abdominal tenderness on palpation. 

**Figure 1:**
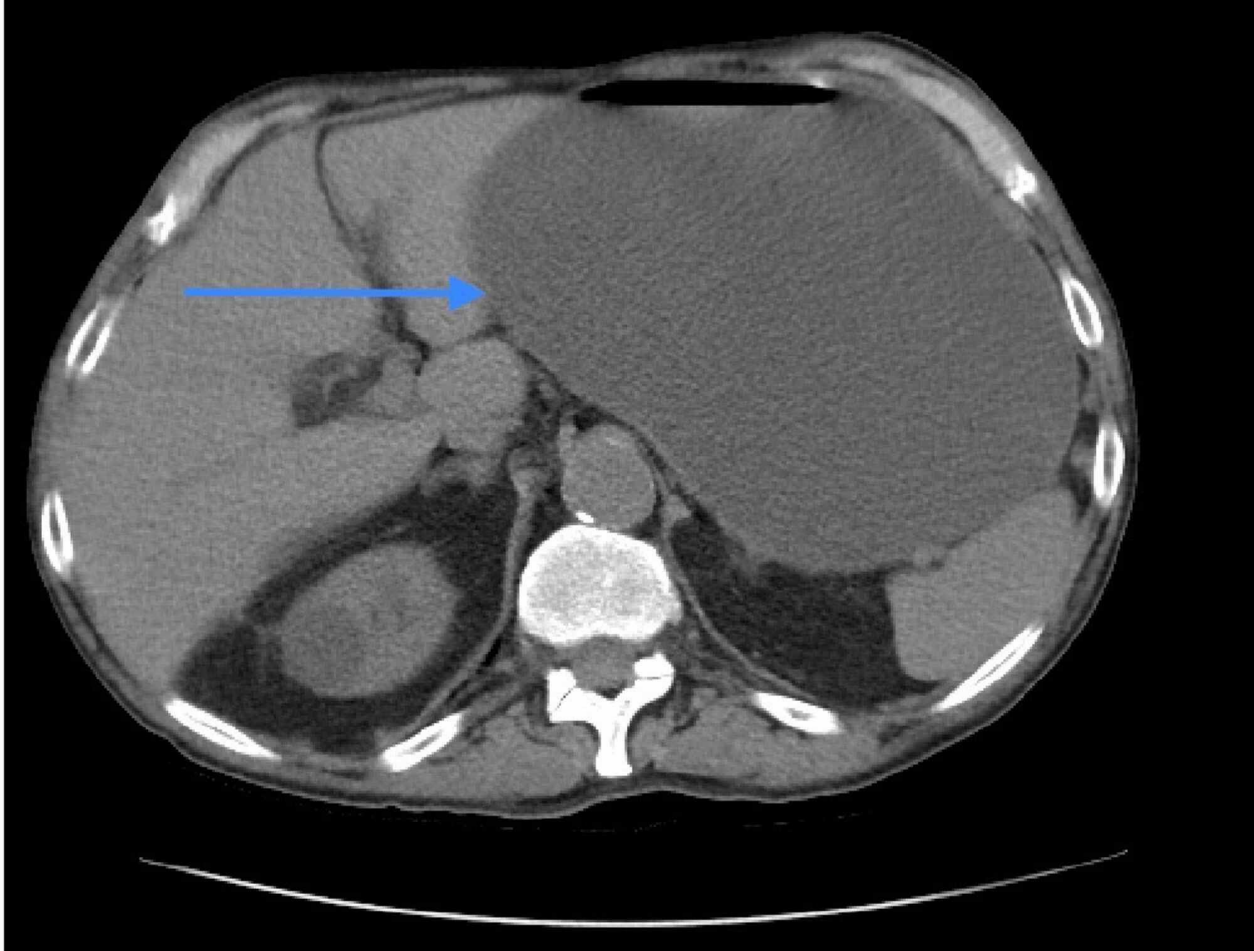
Axial view abdominal CT scan without IV contrast showing a markedly distended stomach

Labs on admission were significant for leukocytosis of 14.4 (reference range, 4.5 - 11 x 10^3^/μl), potassium 2.5 (reference range, 3.5 - 5.3 mmol/L), bicarbonate of 43 (reference range, 23 - 32 mmol/L), anion gap of 20 (reference range 5 - 15 mmol/L), creatinine 2.1 (reference range, 0.6 - 1.4 mg/dL), calcium 12.4 (reference range, 8.6 - 10.3 mg/dL) and lipase of 849 (reference range, 13 - 60 U/L). Cancer antigen 19-9 (CA 19-9) was normal. Liver enzymes were within normal range. Given his acute kidney injury, computed tomography was ordered only with oral contrast. The study revealed massive gastric distention concerning for outlet obstruction (Figure 1). A questionable mass in the head of the pancreas was noted but not well defined due to the lack of intravenous contrast. In addition, there was a pancreatic duct dilation to 5 mm. Initial management focused on gastric decompression with a nasogastric tube. A diagnostic esophagogastroduodenoscopy was performed, showing a dilated, fluid-filled stomach and severe stenosis at the level of the second portion of the duodenum, which could not be traversed. 

**Figure 2:**
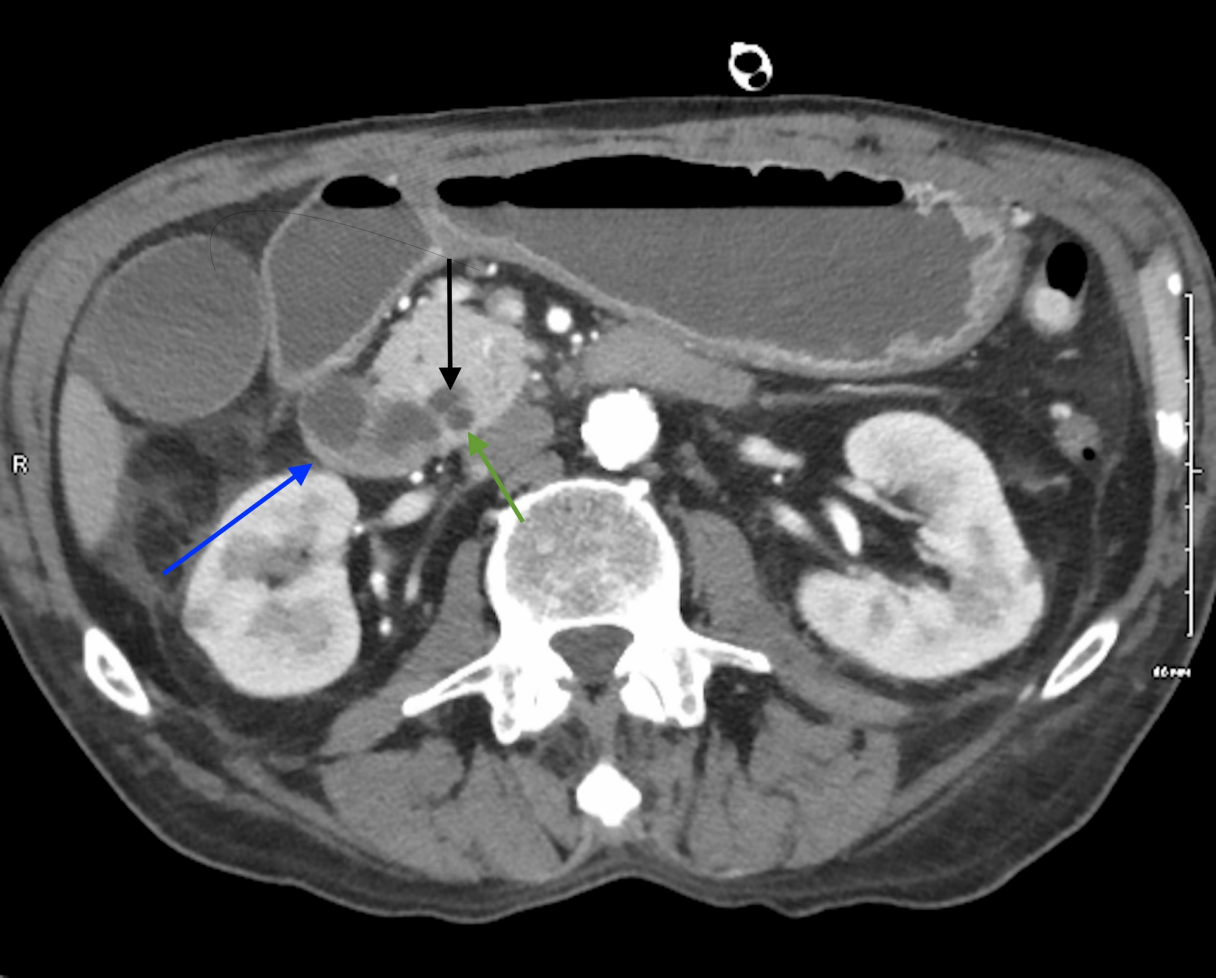
Axial view abdominal CT scan in the arterial phase Blue arrow: showing a multicystic mass; Black arrow: showing common bile duct measuring 5 mm; Green arrow: showing main pancreatic duct measuring 6 mm.

**Figure 3:**
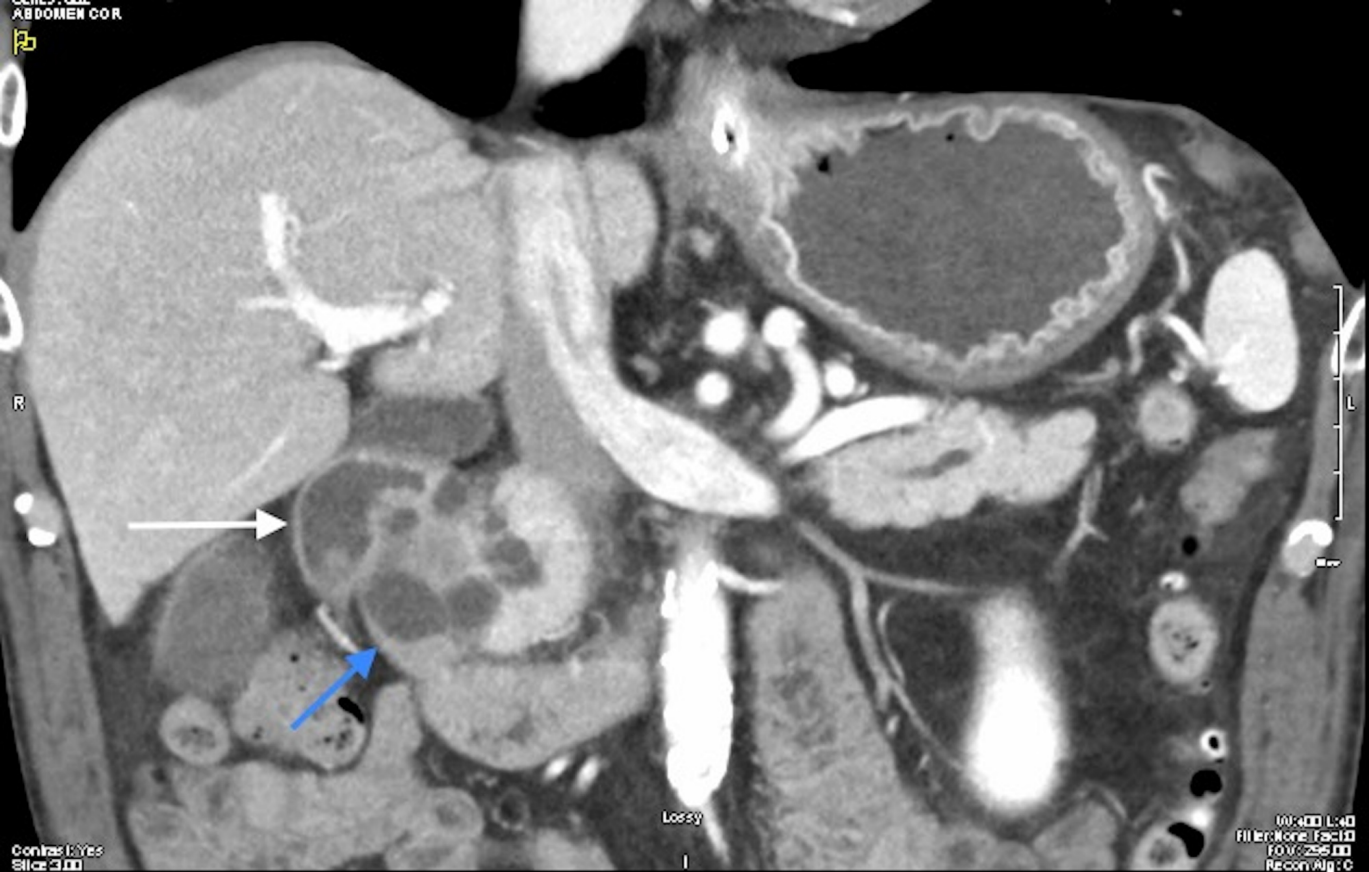
Coronal view abdominal CT scan with IV contrast in arterial phase White arrow: showing second part of duodenum; Blue arrow: cystic mass compressing duodenum.

**Figure 4:**
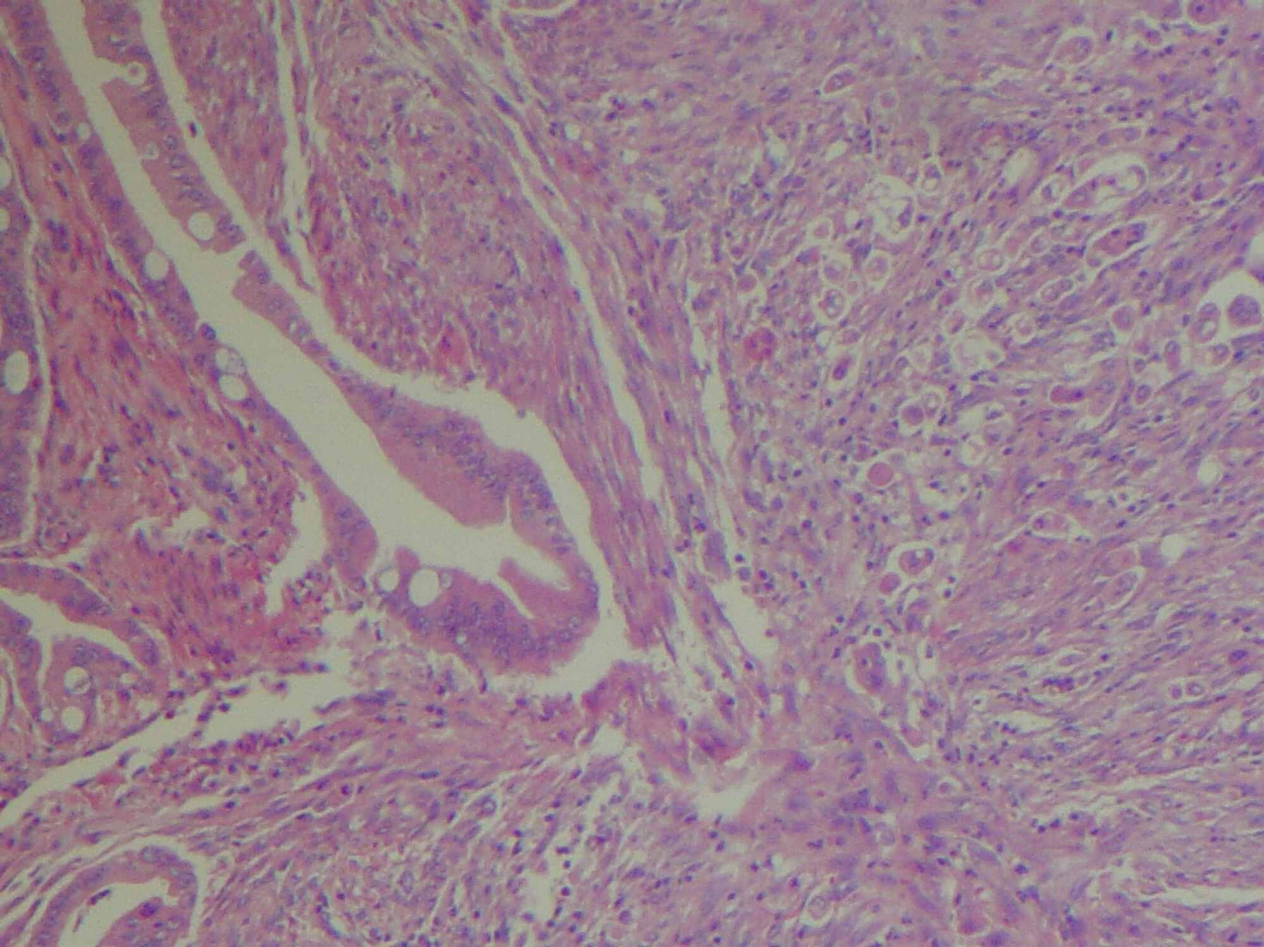
Histology slide of the tumor showing invasive carcinoma, well-differentiated on left, moderately differentiated on right

**Figure 5:**
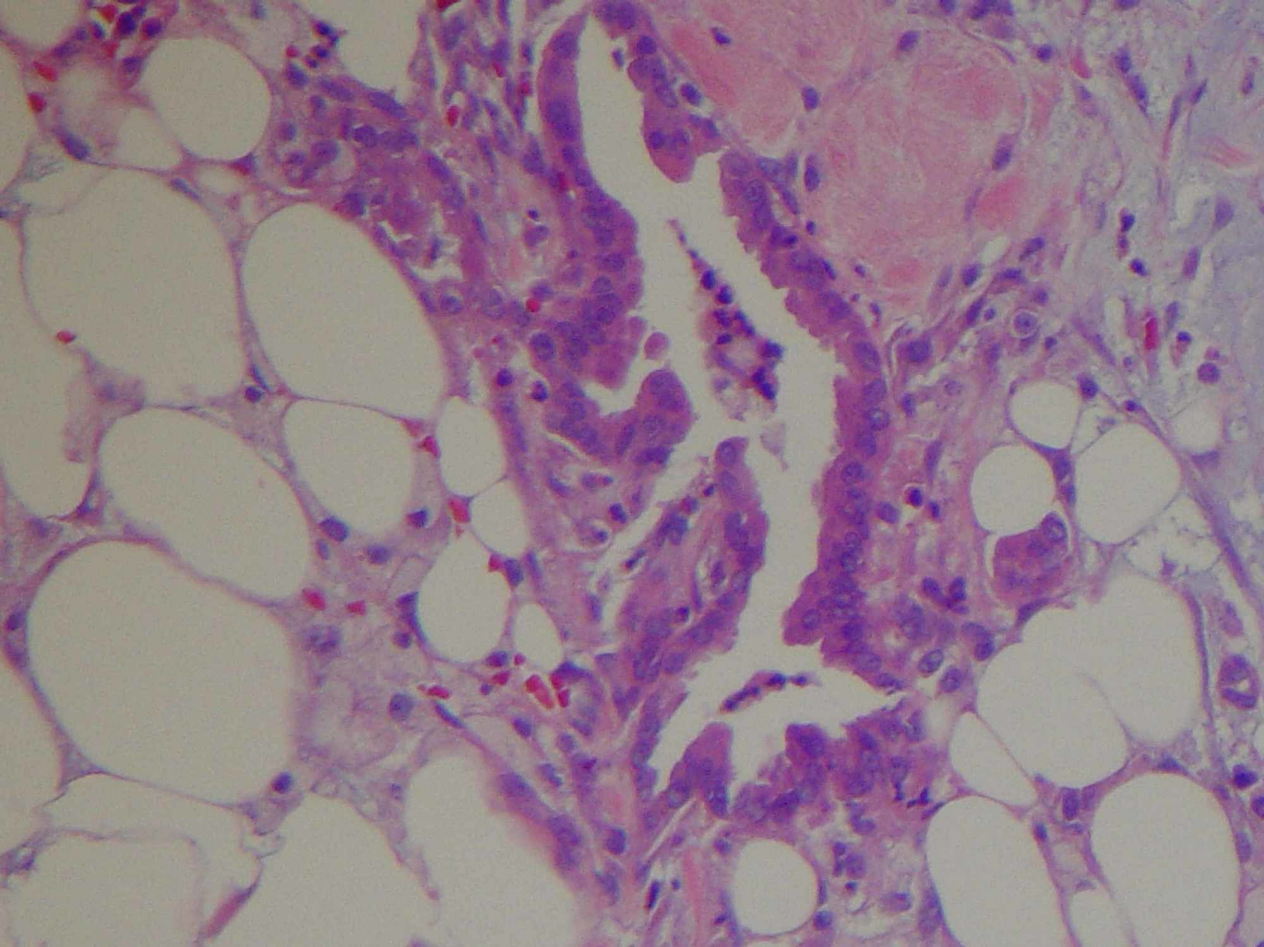
Invasive carcinoma in peripancreatic adipose tissue

**Figure 6:**
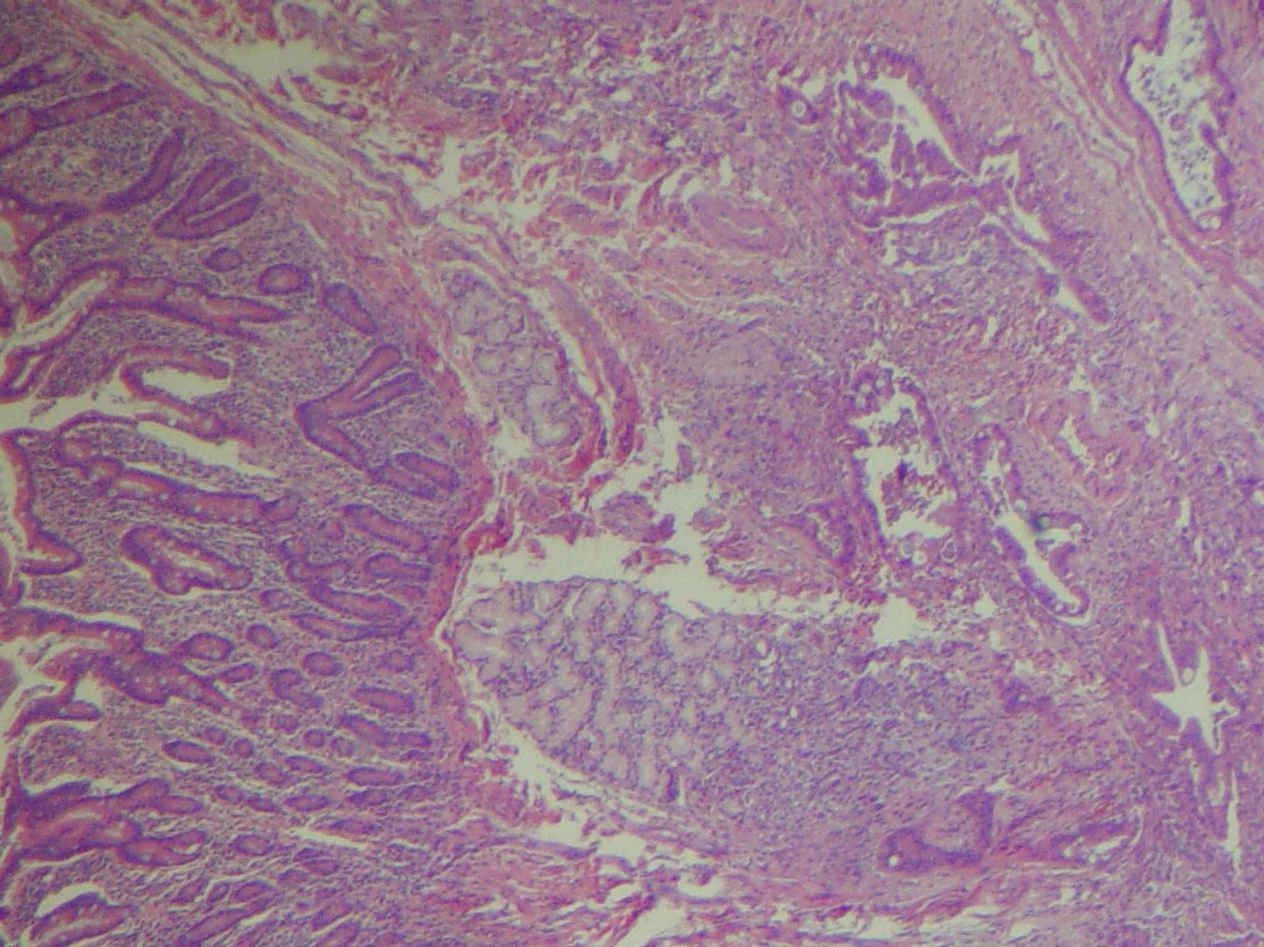
Duodenal tissue on left invaded by tumor on right

**Figure 7:**
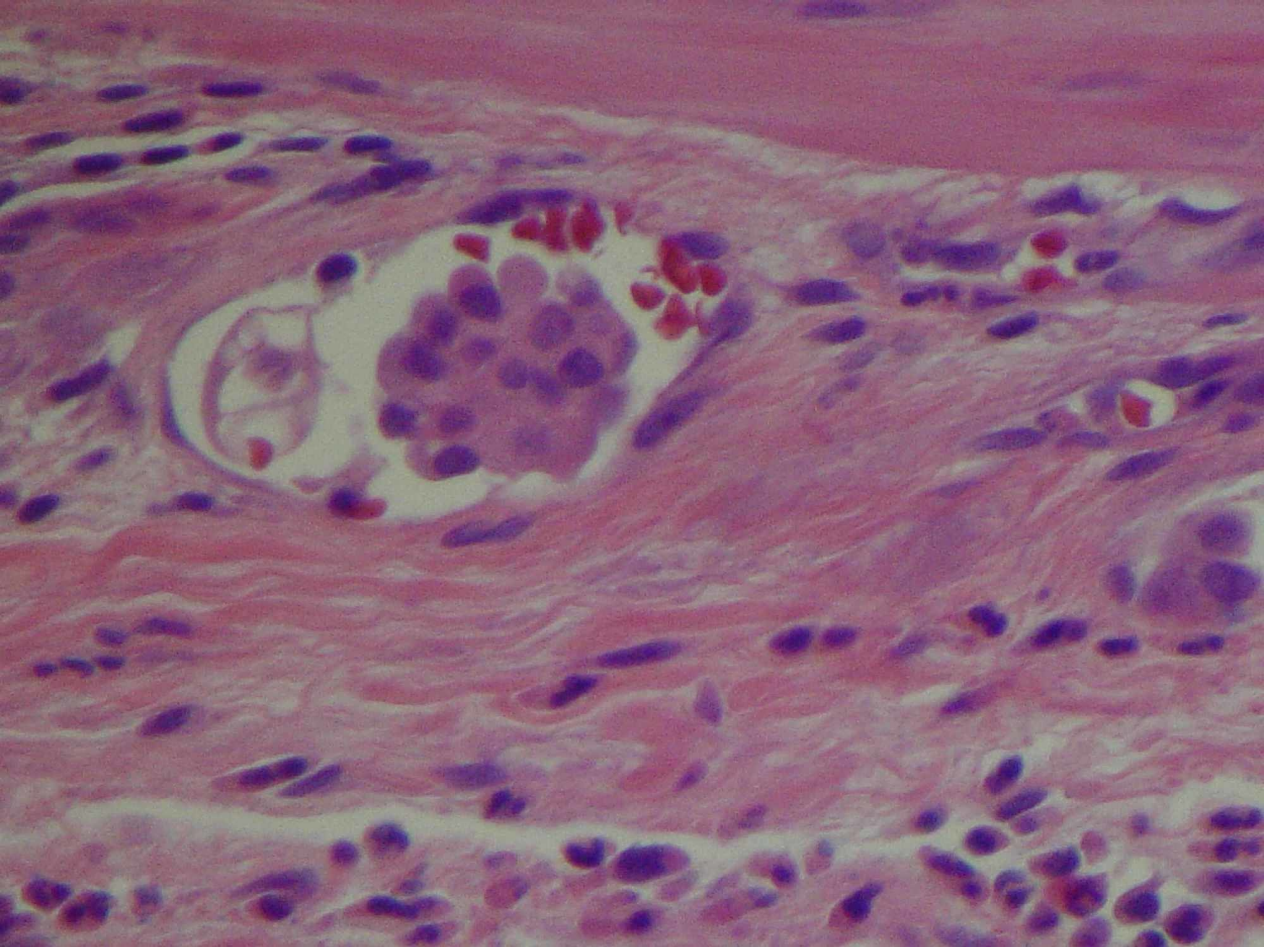
Lymphovascular invasion

After normalization of his kidney function with hydration, a pancreatic protocol enhance contrast CT was done. This showed a multi-cystic mass that extends into the submucosal wall of the duodenum (Figures 2-3). The main pancreatic duct was dilated to 6 mm in the head of the pancreas. Subsequently, an endoscopic ultrasound was done which demonstrated severe stenosis in the second part of the duodenum and an anechoic multi-cystic lesion in the pancreatic head measuring 3.3 x 2.0 cm in cross-sectional diameter with few thick septations. Fine needle aspiration removed 3 mL of thin, yellow-tinged fluid. Carcinoembryonic level of cystic fluid was significantly elevated 575 ng/ml. Amylase was 109 U/L. Cytology was positive for adenocarcinoma. Following cytological confirmation, open pancreaticoduodenectomy was performed. Multiple enlarged hepatic lymph nodes were noted, these were excised and sent for pathology. Pathologic evaluation interestingly revealed ampullary adenocarcinoma of pancreaticobiliary origin with focal signet ring morphology measuring 1.5 x 1.3 x 1 cm in size (Figure 4). The tumor was noted to be invading superficially into pancreatic parenchyma and extending into peripancreatic and periduodenal tissue (Figures 5-6). Lymphophovascular and perineural invasion were identified and 8 to 23 of peripancreatic lymph nodes and one paraesophageal lymph nodes were positive for malignancy (Figure 7). There was no involvement of celiac axis, superior mesenteric artery or common hepatic artery. The tumor was deemed to be T3bN2M0 based on pathological tumor-node-metastasis (pTNM) classification of American Joint Committee for Cancer (AJCC) 8th edition.

Post-operative course was complicated by rising liver enzymes and hepatic artery injury was suspected. Computed tomography angiography (CTA) revealed large irregular hyperattenuating area within the left lobe of the liver that may represent hepatic ischemia. Subsequently, the patient underwent mesenteric angiogram showing hepatic artery thrombosis and subsequently, thrombolysis was performed. Afterwards, the patient had an uncomplicated hospital course and was discharged with close surgical and oncology follow up for chemotherapy evaluation. 

## Discussion

The ampulla of Vater is the point of confluence where the common bile duct and pancreatic duct drain prior to emptying into the duodenum. In the majority of individuals, it serves as the single conduit to the duodenum, and in roughly 42% of individuals, the ampulla drains the common bile duct separate from the pancreatic duct. The area surrounding the ampulla is known as the periampullary region, which is defined as the area within two centimeters of the ampulla of Vater. This is an important anatomical landmark as multiple structures (including the distal common bile duct, second portion of the duodenum, head of the pancreas, and the ampulla itself) exist within this complex anatomic area, all of which can harbor distinct pathology. In terms of malignancy, periampullary cancers account for 5% of all gastrointestinal cancers, however, ampullary cancer itself is a rare entity. Ampullary cancer makes up only 0.2% of all gastrointestinal tumors [1]

Ampullary adenocarcinoma may have early clinical findings that are non-specific such as abdominal pain, lethargy, and weight loss. The early biliary obstruction that clinically manifests as jaundice can prompt a more detailed workup that can result in earlier diagnosis and treatment with surgery and chemotherapy [1].

It has been shown in prior literature that ampullary cancer, as well as other malignancies originating from the periampullary region, can be considered when cross-sectional imaging shows a “double duct sign” in the setting of mass lesion. This finding denotes simultaneous dilatation of the common bile duct and pancreatic duct and has been found in 52% of patients with ampullary adenocarcinoma, and 62% of patients with pancreatic adenocarcinoma [6]. In our case, Following the pancreaticoduodenectomy, a more detailed pathological review of the tissue revealed the final diagnosis of ampullary adenocarcinoma with signet morphology, and the pancreas and bile ducts were spared with pathological review negative for malignancy. 

The majority of ampullary cancers are adenocarcinomas, with a smaller diversity of tumors being papillary, adenosquamous or mucinous [7]. Furthermore, ampullary adenocarcinomas can be divided into two subtypes based on their tissue of origin: intestinal and pancreaticobiliary. Intestinal subtypes express immunohistostaining positive for cytokeratin 20 (CK20) and the caudal homeobox gene transcription factor 2 (CDX2), whereas, pancreaticobiliary subtype, that differentiates from intestinal subtype with immunohistostaining being positive for CK7 and negative for CK20 and CDX2 [8]. It has been shown that the prognosis differs with each subtype, with better outcomes observed with intestinal variant [8]. Despite the difference in prognosis, the best chance of cure is pancreaticoduodenectomy, which is the standard of treatment for either subtype [8].

## Conclusions

Ampullary adenocarcinoma is a rare tumor that most commonly present with jaundice and is detected on radiologic imaging. Initial evaluation requires endoscopic ultrasound with fine needle biopsy. The tumor can be divided into two subtypes, intestinal or pancreaticobiliary based on immunohistochemical staining. Surgery is the mainstay of management with excellent prognosis.
